# Ionization cross sections for collisions between fully stripped ions and ground state hydrogen atoms using the quasi-classical trajectory Monte Carlo method

**DOI:** 10.1038/s41598-026-37732-3

**Published:** 2026-02-17

**Authors:** Iman Ziaeian, Károly Tőkési

**Affiliations:** 1 Physics and Accelerators Research School, Nuclear Science and Technology Research Institute, AEOI, P.O. Box 1439951113, Tehran, Iran; 2 2HUN-REN Institute for Nuclear Research (ATOMKI), 4026 Debrecen Bem tér 18/c, Debrecen, Hungary; 3 HUN-REN Centre for Energy Research, 1121 Budapest, Hungary

**Keywords:** Classical trajectory Monte Carlo model, Quasi-classical trajectory Monte Carlo model, Ionization cross sections, Atomic and molecular physics, Atomic and molecular collision processes

## Abstract

We present ionization cross sections for collisions between fully stripped ions and ground state hydrogen atoms. In these calculations, we employ the standard three-body classical trajectory Monte Carlo (CTMC) and quasi-classical trajectory Monte Carlo (QCTMC) models, focusing on projectile energies ranging from 10 to 1000 keV/amu. The cross sections are analyzed for projectile impacts of H^+^, He^2+^, Li^3+^, Be^4+^, B^5+^, C^6+^, N^7+^, and O^8+^. We found that our QCTMC model yields higher ionization cross sections compared to the CTMC results, particularly at lower impact energies. Additionally, our QCTMC results are in good agreement with both quantum–mechanical findings and experimental data.

## Introduction

Recently, ionic impurities with nuclear charges have been found in the core of thermonuclear experimental reactors^1,2^. Due to the high temperature and density in the core, these impurities are fully stripped ions. One practical method for heating the plasma is to inject an energetic neutral hydrogen beam into it. However, the interaction between impurity ions and hydrogen atoms causes the plasma to cool. The ionization that occurs during collisions between hydrogen atoms and bare particles is one of the most significant processes in atomic physics, as it involves substantial energy transfer. Additionally, ionization by high-energy projectile ions is an intriguing topic, particularly concerning the stopping of neutral beam atoms. Therefore, accurate knowledge of ionization cross sections for these systems is crucial in fusion research.

The single ionization cross sections for collisions of fully stripped ions with nuclear charges and H (1 s) have been studied using various quantum–mechanical models, including semiclassical atomic orbital close-coupling (SC-AOCC)^3^, adiabatic superpromotion^4^, atomic orbital close-coupling (AOCC)^5–8^, continuum distorted wave-Eikonal initial state approximation^9,10^, two-center momentum space discretization^11^, two-center basis generator (TC-BGM)^12–16^, and two-center wave-packet convergent close-coupling (WP-CCC)^17–19^ methods. The ionization cross sections for He^2+^, Li^3+^, and C^6+^colliding with excited hydrogen atoms have been reported in Refs.^20,21^. Regarding classical models, Jorge et al*.*^22^ have performed calculations for collisions between C^6+^ and N^7+^, as well as atomic hydrogen, using the microcanonical classical trajectory Monte Carlo (CTMC) method in the 5–500 keV/amu projectile energy range. Hardie and Olson^23^ improved the CTMC cross section by utilizing the hydrogenic CTMC method (H-CTMC), which involves a superposition of eight microcanonical distributions with varying energies. The classical approach for bare ion impacts on H, based on the impact parameter CTMC method, has also been discussed in Refs.^24,25^. In recent work, Hill et al*.*^26^ reviewed and compared theoretical methods for calculating ionization cross sections relevant to neutral beam modeling in fusion plasmas, including H + Be^4+^ and H + H^+^. Experimental data for the ionization of H(1 s) by H^1+^, He^2+^, and Li^3+^ impacts have been measured and reported in Refs.^27–32^.

In many cases, quantum–mechanical calculations are very complicated and unfeasible. Therefore, as an alternative calculation scheme and due to the simplicity of calculations, classical models have been developed. The classical trajectory Monte Carlo (CTMC) method is a well-known nonperturbative technique used for modeling atomic collisions and calculating various cross sections^33–48^. However, due to the absence of quantum features in the standard CTMC model, it cannot accurately describe cross sections, particularly at lower impact energies where quantum mechanical effects may dominate. To address this limitation, the quasi-classical trajectory Monte Carlo (QCTMC) model was developed.

In this context, a model potential was introduced by Kirschbaum and L. Wilet to mimic quantum effects^49^, such as the Heisenberg uncertainty principle, for multi-electron systems. This effective potential enforces the Heisenberg uncertainty principle, where *r* and *p* represent the distance and momentum of an electron with respect to a nucleus, respectively, and a constant is involved. This potential helps prevent the collapse of multi-electron systems. Prior to this development, even two-electron systems were unstable when considering electron–electron interactions. Classically, the repulsive Coulomb interaction between electrons is responsible for this instability, leading to autoionization of the system. The QCTMC model has recently been used to calculate the cross sections of atomic collision systems^50–55^.

In this work, we present the ionization cross sections for bare ions A^Z+^, colliding with H(1 s). We performed our calculations using both three-body classical trajectory Monte Carlo and three-body quasi-classical trajectory Monte Carlo models. Our calculated cross sections are compared with available quantum–mechanical results and experimental data.

## Theory

In the three-body quasi-classical trajectory Monte Carlo model, a correction term is added to the standard classical Hamiltonian of the collision system using a model potential proposed by Kirschbaum and Wilet^49^ to mimic the Heisenberg uncertainty principle. Consequently, the Hamiltonian of the atomic collision system is defined as follows:

1 $${H}_{QCTMC}={H}_{0}+{V}_{H},$$

where H_0_ is the standard Hamiltonian that includes the total kinetic energy of all bodies, as well as the Coulomb potential terms for all pairs of electrons and between the nucleus and electrons. The Heisenberg correction term is

2 $$V_{H} = \sum\nolimits_{{n = a.b}} {\sum\nolimits_{{i = 1}}^{N} {f\left( {r_{{ni}} {\mathrm{~}}.p_{{ni}} ;\xi _{H} {\mathrm{~}}.{\mathrm{~}}\alpha _{H} } \right),} }$$

where *a* and *b* denote the nuclei and the *i* index the electrons. Also, ${r}_{\lambda \nu }={r}_{\nu }-{r}_{\lambda }$ and relative momenta are:

3 $${p}_{\lambda \nu }=\frac{{m}_{\lambda }{p}_{\nu }-{m}_{\nu }{p}_{\lambda }}{{m}_{\lambda }+{m}_{\nu }}.$$

The Heisenberg correction function is defiend as^49^:

4 $$f\left({r}_{\lambda \nu } .{p}_{\lambda \nu };{\xi }_{H} . {\alpha }_{H}\right)=\frac{{{\xi }_{H}}^{2}}{4{\alpha }_{H}{r}_{\uplambda \nu }^{2}{\mu }_{\lambda \nu }}exp\left\{{\alpha }_{H}\left[1-{\left(\frac{{r}_{\lambda \nu }{p}_{\lambda \nu }}{{\xi }_{H}}\right)}^{4}\right]\right\},$$

where $\mu$ is the reduced mass between two bodies. Additionally, ${\alpha }_{H}$ and ${\xi }_{H}$ are the adjustable hardness and dimensionless parameters, respectively. In our calculations, we used ${\alpha }_{H}$= 3.5 and ${\xi }_{H}$= 0.9354, parameters relevant for the hydrogen target^51^. We also considered the Heisenberg correction term between the target electron and both the target nucleus and the projectile as follows:

5 $$f\left({\overrightarrow{r}}_{ep} .{\overrightarrow{P}}_{pe};{\varepsilon }_{H} . {\alpha }_{H}\right)=\frac{{{\xi }_{H}}^{2}}{4{\alpha }_{H}{r}_{pe}^{2}{\mu }_{pe}}exp\left\{{\alpha }_{H}\left[1-{\left(\frac{{r}_{pe}{P}_{pe}}{{\xi }_{H}}\right)}^{4}\right]\right\}$$

6 $$f\left({\overrightarrow{r}}_{Te} .{\overrightarrow{P}}_{Te};{\varepsilon }_{H} . {\alpha }_{H}\right)=\frac{{{\xi }_{H}}^{2}}{4{\alpha }_{H}{r}_{Te}^{2}{\mu }_{Te}}exp\left\{{\alpha }_{H}\left[1-{\left(\frac{{r}_{Te}{P}_{Te}}{{\xi }_{H}}\right)}^{4}\right]\right\},$$

where *T*, *p*, and *e* represent the target, projectile, and electron, respectively.

The equations of motion, which incorporate Hamiltonian mechanics and the Heisenberg correction term, are as follows:

7 $$\begin{aligned} \mathop {\vec{P}}\limits_{e} = & - \frac{\delta H}{{\delta \vec{r}_{e} }} = - \frac{{Z_{p} Z_{e} }}{{\left| {\vec{r}_{p} - \vec{r}_{e} } \right|^{3} }}\left( {\vec{r}_{p} - \vec{r}_{e} } \right) + \frac{{Z_{e} Z_{T} }}{{\left| {\vec{r}_{e} - \vec{r}_{T} } \right|^{3} }}\left( {\vec{r}_{e} - \vec{r}_{T} } \right) \\ & + \left( {\frac{{\xi_{H}^{2} }}{{2\alpha_{H} \mu_{Te} \left| {\vec{r}_{e} - \vec{r}_{T} } \right|^{4} }} + \frac{{\vec{P}_{Te}^{4} }}{{\xi_{H}^{2} \mu_{Te} }}} \right)e^{{\alpha_{H} \left[ {1 - \left( {\frac{{\left| {\vec{r}_{e} - \vec{r}_{T} } \right|\left| {\vec{P}_{Te} } \right|}}{{\xi_{H} }}} \right)^{4} } \right]}} \left( {\vec{r}_{e} - \vec{r}_{T} } \right) \\ & + \left( {\frac{{\xi_{H}^{2} }}{{2\alpha_{H} \mu_{pe} \left| {\vec{r}_{e} - \vec{r}_{p} } \right|^{4} }} + \frac{{\vec{P}_{pe}^{4} }}{{\xi_{H}^{2} \mu_{pe} }}} \right)e^{{\alpha_{H} \left[ {1 - \left( {\frac{{\left| {\vec{r}_{e} - \vec{r}_{p} } \right|\left| {\vec{P}_{pe} } \right|}}{{\xi_{H} }}} \right)^{4} } \right]}} \left( {\vec{r}_{p} - \vec{r}_{e} } \right) \\ \end{aligned}$$

8 $${\overrightarrow{\dot{r}}}_{e}=\frac{\partial {H}_{FMD}}{\partial {\overrightarrow{P}}_{e}}={N}_{e}{\overrightarrow{P}}_{e}-\frac{{N}_{e}{\left|{\overrightarrow{r}}_{e}-{\overrightarrow{r}}_{T}\right|}^{2}{\left|{\overrightarrow{P}}_{Te}\right|}^{2}}{{{\xi }_{H}}^{2}}{e}^{{\alpha }_{H}\left[1-{\left(\frac{\left|{\overrightarrow{r}}_{e}-{\overrightarrow{r}}_{T}\right|\left|{\overrightarrow{P}}_{Te}\right|}{{\xi }_{H}}\right)}^{4}\right]}{\overrightarrow{P}}_{Te}+\frac{{N}_{e}{\left|{\overrightarrow{r}}_{p}-{\overrightarrow{r}}_{e}\right|}^{2}{\left|{\overrightarrow{P}}_{pe}\right|}^{2}}{{{\xi }_{H}}^{2}}{e}^{{\alpha }_{H}\left[1-{\left(\frac{\left|{\overrightarrow{r}}_{p}-{\overrightarrow{r}}_{e}\right|\left|{\overrightarrow{P}}_{pe}\right|}{{\xi }_{H}}\right)}^{4}\right]}{\overrightarrow{P}}_{pe}$$

9 $${\dot{\overrightarrow{P}}}_{T}=-\frac{\delta H}{\delta {\overrightarrow{r}}_{T}}=-\frac{{Z}_{p}{Z}_{T}}{{\left|{\overrightarrow{r}}_{p}-{\overrightarrow{r}}_{T}\right|}^{3}}\left({\overrightarrow{r}}_{p}-{\overrightarrow{r}}_{T}\right)-\frac{{Z}_{e}{Z}_{T}}{{\left|{\overrightarrow{r}}_{e}-{\overrightarrow{r}}_{T}\right|}^{3}}\left({\overrightarrow{r}}_{e}-{\overrightarrow{r}}_{T}\right)+ \left(\frac{{{\xi }_{H}}^{2}}{2{\alpha }_{H}{\mu }_{Te}{\left|{\overrightarrow{r}}_{e}-{\overrightarrow{r}}_{T}\right|}^{4}}+\frac{{\overrightarrow{P}}_{Te}^{4}}{{{\xi }_{H}}^{2}{\mu }_{Te}}\right){e}^{{\alpha }_{H}\left[1-{\left(\frac{\left|{\overrightarrow{r}}_{e}-{\overrightarrow{r}}_{T}\right|\left|{\overrightarrow{P}}_{Te}\right|}{{\xi }_{H}}\right)}^{4}\right]}\left({\overrightarrow{r}}_{e}-{\overrightarrow{r}}_{T}\right)$$

10 $${\overrightarrow{\dot{r}}}_{T}=\frac{\partial {H}_{FMD}}{\partial {\overrightarrow{P}}_{p}}={N}_{T}{\overrightarrow{P}}_{T}-\frac{{N}_{T}{\left|{\overrightarrow{r}}_{e}-{\overrightarrow{r}}_{T}\right|}^{2}{\left|{\overrightarrow{P}}_{Te}\right|}^{2}}{{{\xi }_{H}}^{2}}{e}^{{\alpha }_{H}\left[1-{\left(\frac{\left|{\overrightarrow{r}}_{e}-{\overrightarrow{r}}_{T}\right|\left|{\overrightarrow{P}}_{Te}\right|}{{\xi }_{H}}\right)}^{4}\right]}{\overrightarrow{P}}_{Te}$$

11 $${\dot{\overrightarrow{P}}}_{p}=-\frac{\delta {H}_{FMD}}{\delta {\overrightarrow{r}}_{p}}=\frac{{Z}_{p}{Z}_{e}}{{\left|{\overrightarrow{r}}_{p}-{\overrightarrow{r}}_{e}\right|}^{3}}\left({\overrightarrow{r}}_{p}-{\overrightarrow{r}}_{e}\right)+\frac{{Z}_{p}{Z}_{T}}{{\left|{\overrightarrow{r}}_{p}-{\overrightarrow{r}}_{T}\right|}^{3}}\left({\overrightarrow{r}}_{p}-{\overrightarrow{r}}_{T}\right)-\left(\frac{{{\xi }_{H}}^{2}}{2{\alpha }_{H}{\mu }_{pe}{\left|{\overrightarrow{r}}_{e}-{\overrightarrow{r}}_{p}\right|}^{4}}+\frac{{\overrightarrow{P}}_{pe}^{4}}{{{\xi }_{H}}^{2}{\mu }_{pe}}\right){e}^{{\alpha }_{H}\left[1-{\left(\frac{\left|{\overrightarrow{r}}_{e}-{\overrightarrow{r}}_{p}\right|\left|{\overrightarrow{P}}_{pe}\right|}{{\xi }_{H}}\right)}^{4}\right]}\left({\overrightarrow{r}}_{p}-{\overrightarrow{r}}_{e}\right)$$

12 $${\overrightarrow{\dot{r}}}_{p}=\frac{\partial {H}_{FMD}}{\partial {\overrightarrow{P}}_{p}}={N}_{p}{\overrightarrow{P}}_{p}+\frac{{N}_{p}{\left|{\overrightarrow{r}}_{p}-{\overrightarrow{r}}_{e}\right|}^{2}{\left|{\overrightarrow{P}}_{pe}\right|}^{2}}{{{\xi }_{H}}^{2}}{e}^{{\alpha }_{H}\left[1-{\left(\frac{\left|{\overrightarrow{r}}_{p}-{\overrightarrow{r}}_{e}\right|\left|{\overrightarrow{P}}_{pe}\right|}{{\xi }_{H}}\right)}^{4}\right]}{\overrightarrow{P}}_{pe}.$$

In the classical approaches, the classical principal quantum number (*n*_*c*_) is defined by

13 $${n}_{c}={Z}_{T}{Z}_{e}{\left(\frac{{\mu }_{Te}}{2U}\right)}^{1/2},$$

where ${\mu }_{Te}$ and *U* is the reduced mass of the target nucleus and the target electron, and the electron binding energy. The classical values of *n*_*c*_ are quantized to a specific level *n* if they satisfy the relation^56^:

14 $${\left[\left(n-1\right)\left(n-1/2\right)n\right]}^{1/3}\le {n}_{c}\le {\left[\left(n+1\right)\left(n+\frac{1}{2}\right)n\right]}^\frac{1}{3}.$$

The classical orbital angular momentum quantum number (*l*_*c*_) is given by

15 $${l}_{c}=\sqrt{{{l}_{c}^{x}}^{2}+{{l}_{c}^{y}}^{2}+{{l}_{c}^{z}}^{2}} , \mathrm{with}$$

16 $${l}_{c}^{x}= {m}_{e}\left(x\dot{y}-y\dot{x}\right),\;{ l}_{c}^{y}= {m}_{e}\left(x\dot{z}-z\dot{x}\right),\; {l}_{c}^{z}= {m}_{e}\left(y\dot{z}-z\dot{y}\right),$$

where x, y, and z are the Cartesian coordinates of the electron relative to the nucleus, and $\dot{x}$, $\dot{y}$, and $\dot{z}$ are the corresponding velocities. Since ${l}_{c}$ the distribution is uniform for a given *n* level, the quantal statistical weights are obtained by selecting bin sizes such that^57^:

17 $$l\le \frac{n}{{n}_{c}}{l}_{c}\le l+1,$$

where *l* is the quantum–mechanical orbital angular momentum.

The total cross sections can be calculated by:

18 $$\sigma =\frac{2\pi {b}_{max}}{{T}_{N}}\sum_{j}{b}_{j}^{(i)},$$

and the statistical uncertainty of the cross sections is given by:

19 $$\Delta \sigma =\sigma {\left(\frac{{T}_{N}-{T}_{N}^{(i)}}{{T}_{N}{T}_{N}^{(i)}}\right)}^{\raisebox{1ex}{$1$}\!\left/ \!\raisebox{-1ex}{$2$}\right.},$$

where *T*_*N*_ is the total number of trajectories calculated for impact parameters less than *b*_*max*_, $T_{N}^{(i)}$ is the number of trajectories that satisfy the criteria for the corresponding final channels (electron capture), and *b*_*j*_^*(i)*^ is the actual impact parameter for the trajectory corresponding to electron capture processes.

## Results and discussions

To study the ionization cross sections in A^Z+^ with 1 ≤ *Z* ≤ 8 and a ground-state hydrogen atom (see Eq. 20), we performed calculations in the projectile energy range of 10–1000 keV/amu using both the standard three-body CTMC and QCTMC models.

20 $${\mathrm{A}}^{{{\mathrm{Z}} + }} + {\text{ H}}\left( {{\mathrm{1s}}} \right) \, \to {\text{ A}}^{{{\mathrm{Z}} + }} + {\text{ H}}^{ + } + {\mathrm{e}} - .$$

To increase the accuracy of our calculations, we simulated 5 × 10^6^ individual trajectories in both the CTMC and QCTMC models for each impact energy. Based on a large number of primary histories, the estimated uncertainties (see Eq. 19) of the cross sections in both models are approximately 0.6%. We compared our CTMC and QCTMC results with available quantum–mechanical approaches, including AOCC^5,6^, CDW^9,10^, two-center momentum space discretization^11^, TC-BGM^12^, WP-CCC^17^, the classical H-CTMC model^23^, finite difference^58^, and experimental data^27,28^.

**Fig. 1:**
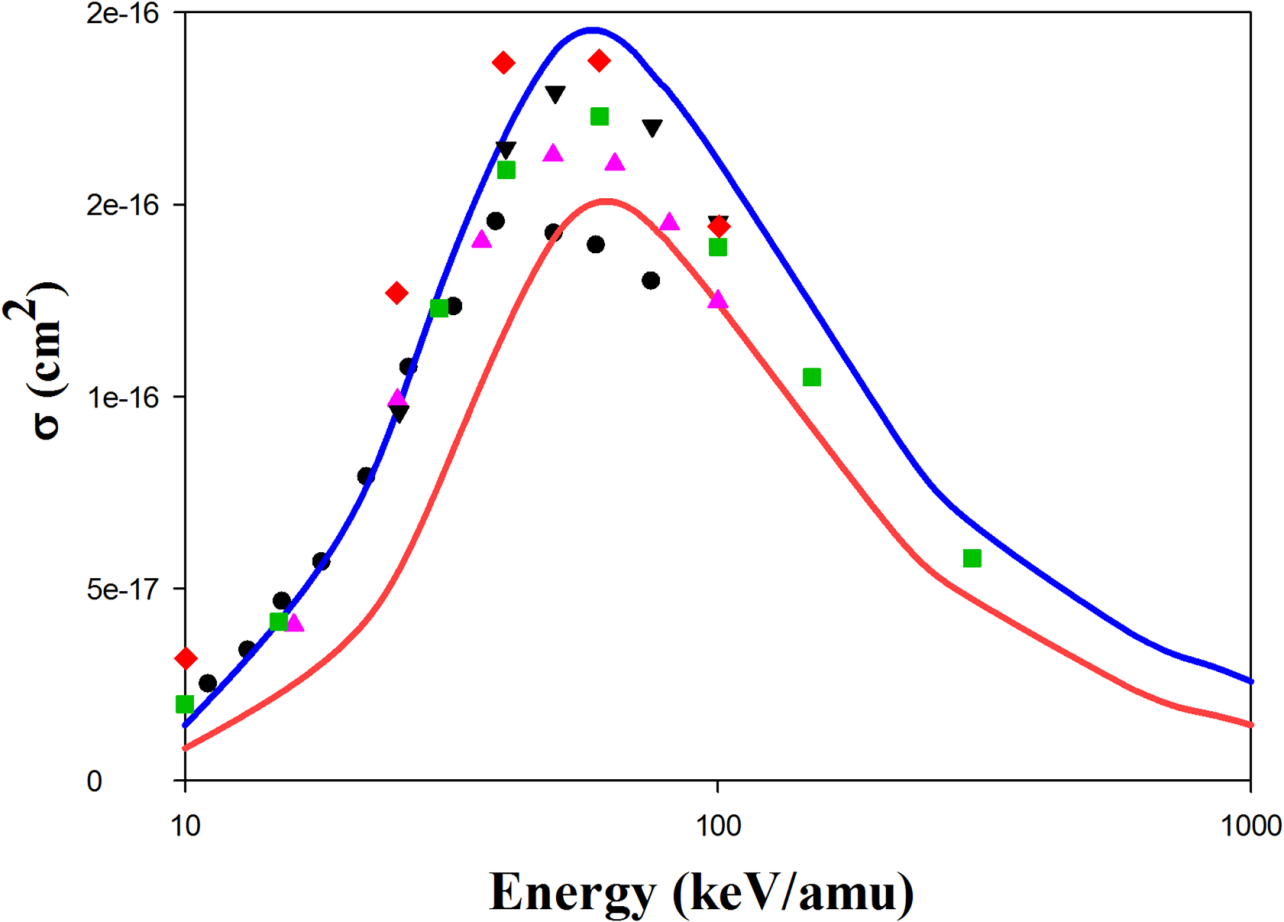
Projectile energy dependent ionization cross sections in H^+^ + H(1s) collisions. Solid red line: present CTMC results, solid blue line: present QCTMC results, pink triangles: SC-AOCC results of Agueny^3^, green square: TC-BGM results of Leung et al.^12^, black inverse triangles: two-center momentum space discretization results of Sidky and Lin^11^, red diamond: finite difference results of Kolakowska et al.^58^, black circles: experimental results of Shah et al.^28^.

Figure 1 represents the ionization cross sections in H^+^  + H(1 s) collisions. The QCTMC model significantly increases the cross-sectional area compared to the CTMC model. Our QCTMC results agree well with the quantum mechanics approaches such as SC-AOCC^3^, TC-BGM^12^, two-center momentum space discretization^11^, and finite difference^58^ at impact energies below 100 keV/amu. Furthermore, the experimental results of Shah et al.^28^ are in good agreement with our QCTMC results at low impact energies.

**Fig. 2:**
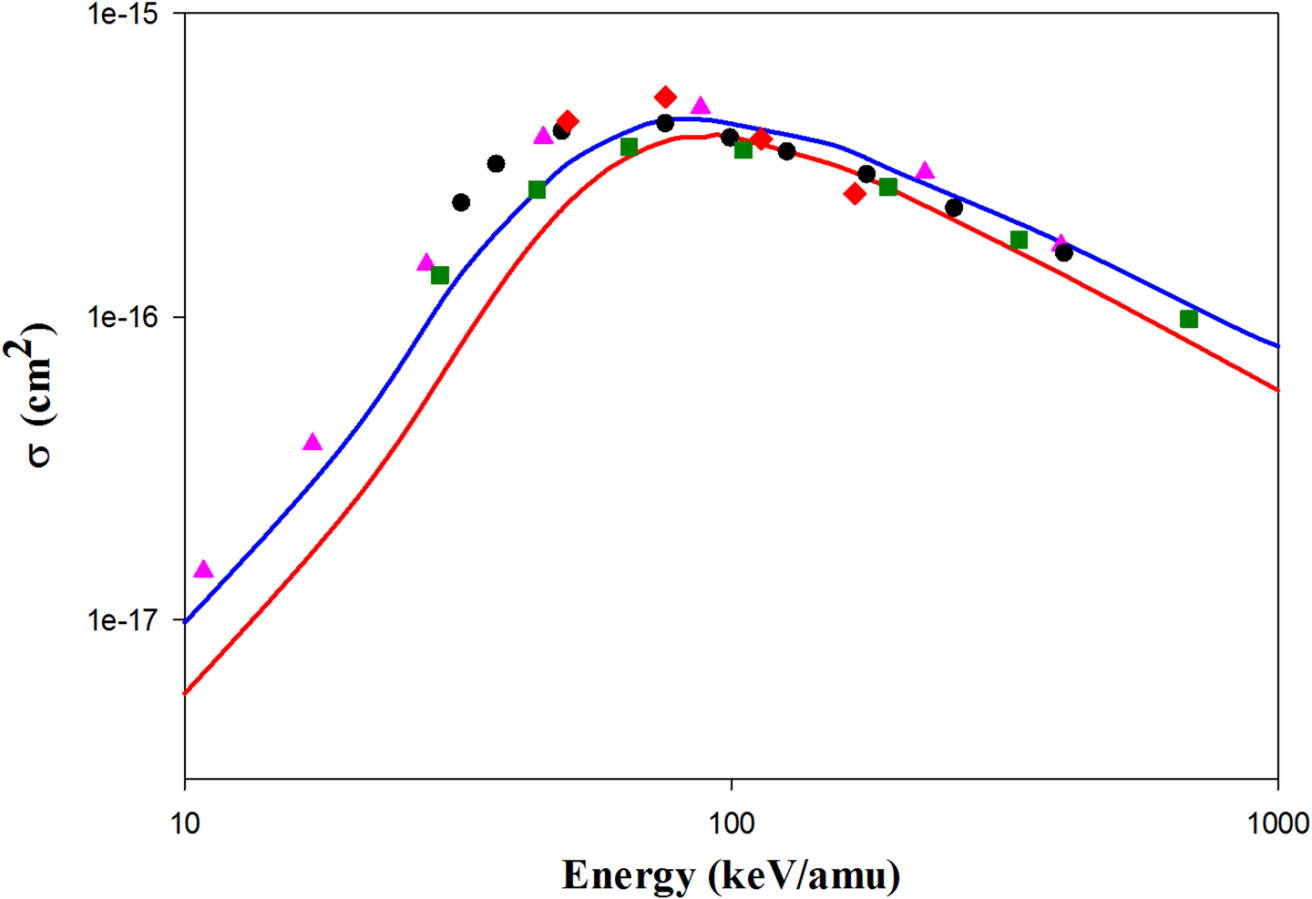
Projectile energy dependent ionization cross sections in He^2+^  + H(1s) collisions. Solid red line: present CTMC results, solid blue line: present QCTMC results, pink triangles: AOCC results of Toshima^5^, green squares: CDW results of Crothers et al*.*^10^, red diamonds: H-CTMC results of Hardie et al*.*^23^, black circles: experimental results of Shah et al*.*^27^.

Figure 2 shows the projectile energy-dependent ionization cross sections in He^2+^  + H(1 s) collisions. We found that the QCTMC model increases the ionization cross sections compared to the CTMC model. Our QCTMC results are in good agreement with the AOCC results of Toshima^5^ at projectile energies between 10 and 1000 keV/amu. Additionally, the CDW results of Crothers et al.^10^ align well with our QCTMC results at intermediate projectile energies, as well as with both the CTMC and QCTMC results at higher impact energies. The experimental data^27^ are closer to the QCTMC results at impact energies between 35 and 100 keV/amu compared to the CTMC results.

**Fig. 3:**
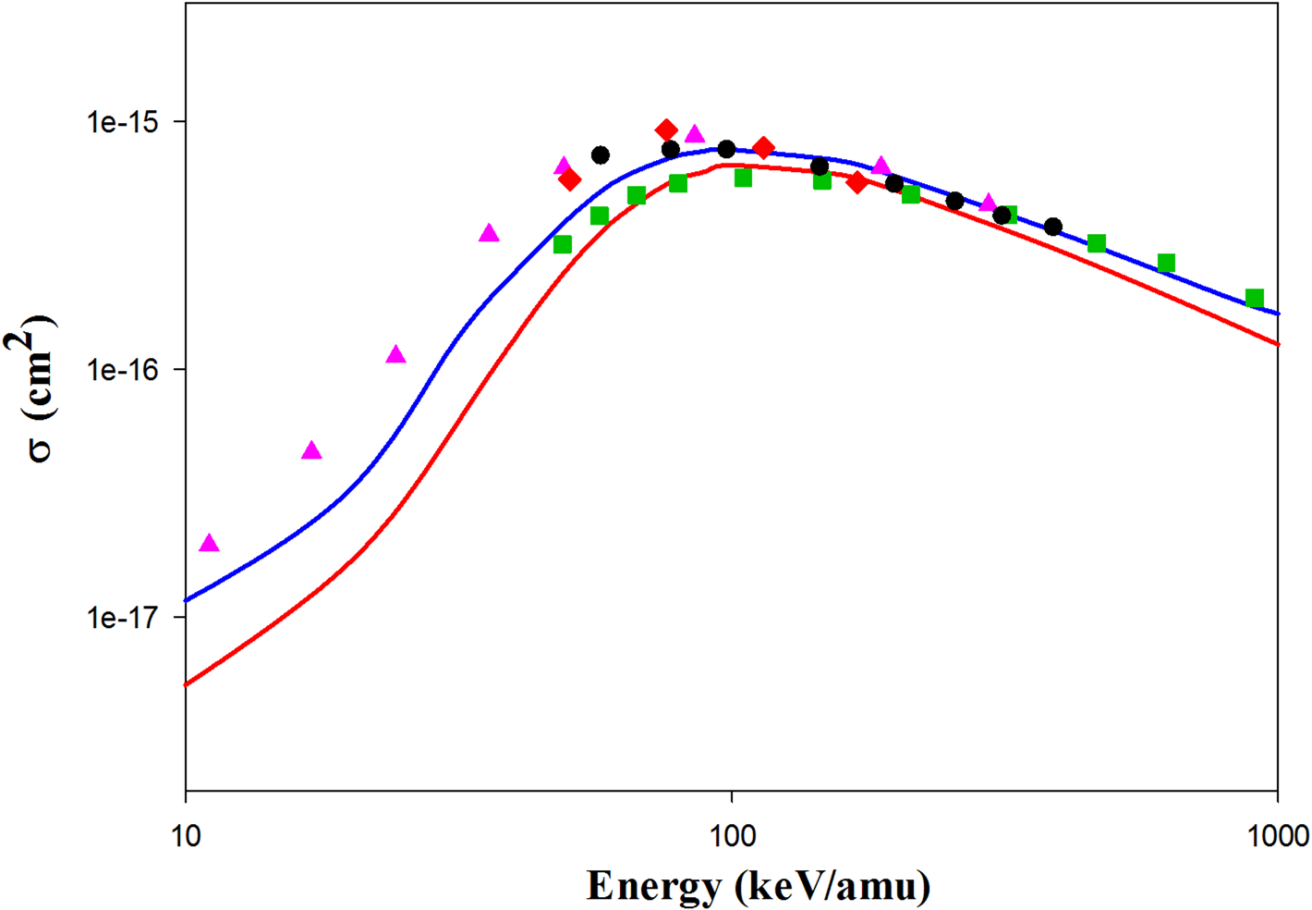
Projectile energy dependent ionization cross sections in Li^3+^  + H(1s) collisions. Solid red line: present CTMC results, solid blue line: present QCTMC results, pink triangles: AOCC results of Toshima^5^, green squares: CDW results of Crothers et al*.*^10^, red diamonds: H-CTMC results of Hardie et al*.*^23^, black circles: experimental results of Shah et al*.*^27^.

Figure 3 shows the ionization cross sections for Li^3+^  + H(1 s) collisions. The QCTMC ionization cross sections align more closely with the AOCC results from Toshima^5^ and the hydrogenic-CTMC results from Hardi et al.^23^ than the current CTMC results do. Additionally, the QCTMC results are in good agreement with the CDW results from Crothers et al.^10^ above 200 keV/amu, and they also correlate well with the experimental data^27^.

**Fig. 4:**
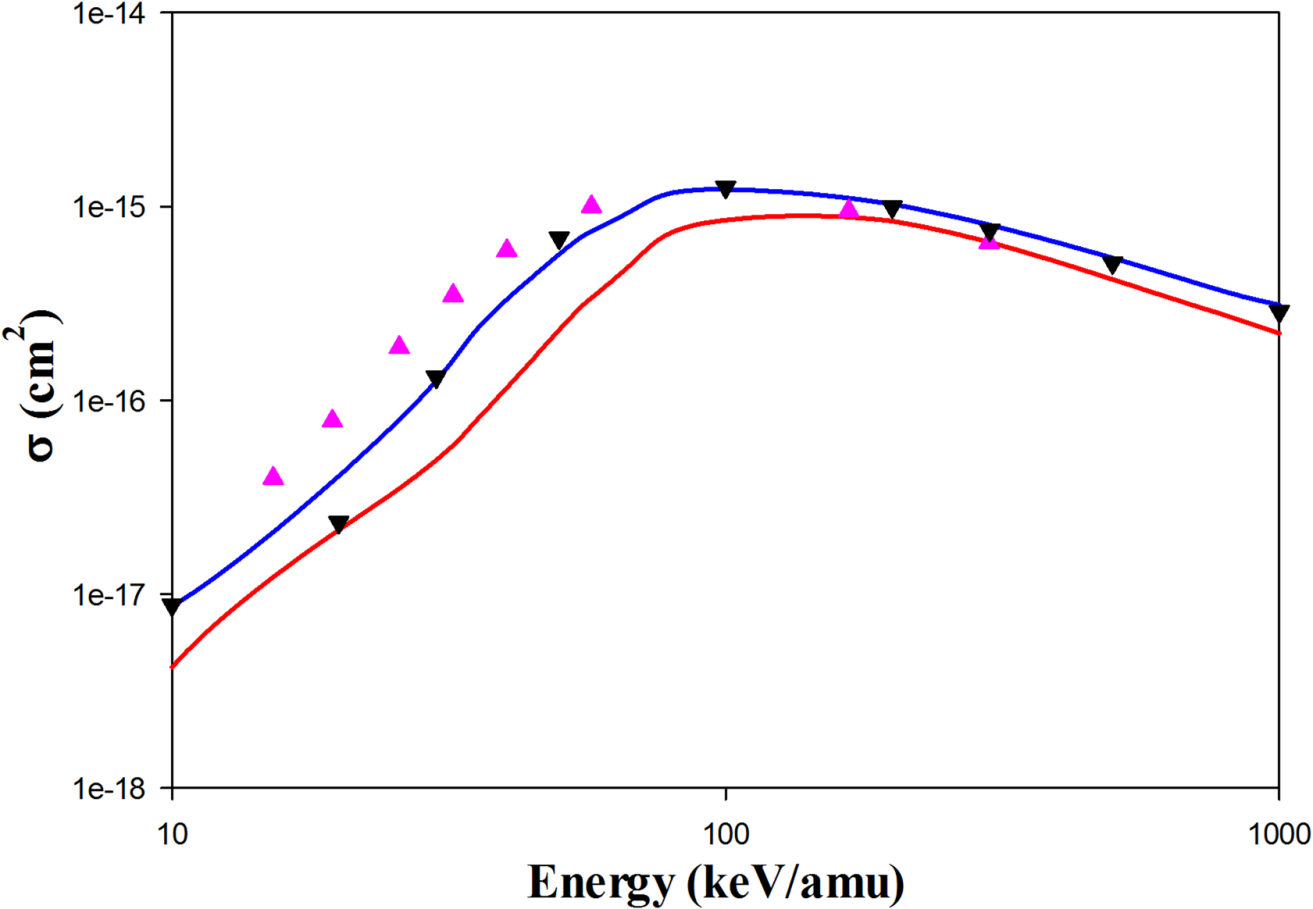
Projectile energy dependent ionization cross sections in Be^4+^  + H(1s) collisions. Solid red line: present CTMC results, solid blue line: present QCTMC results, pink triangles: AOCC results of Toshima^5^, black inverse triangles: WP-CCC results of Antonio et al.^17^.

Figure 4 represents the ionization cross sections in Be^4+^  + H(1s) collisions. The QCTMC model improves the ionization cross sections across the entire range of impact energies. The WP-CCC results from Antonio et al.^17^ align well with our QCTMC results. Furthermore, the AOCC results from Toshima^5^ are closer to our QCTMC results than the CTMC ones at impact energies below 100 keV/amu.

**Fig. 5:**
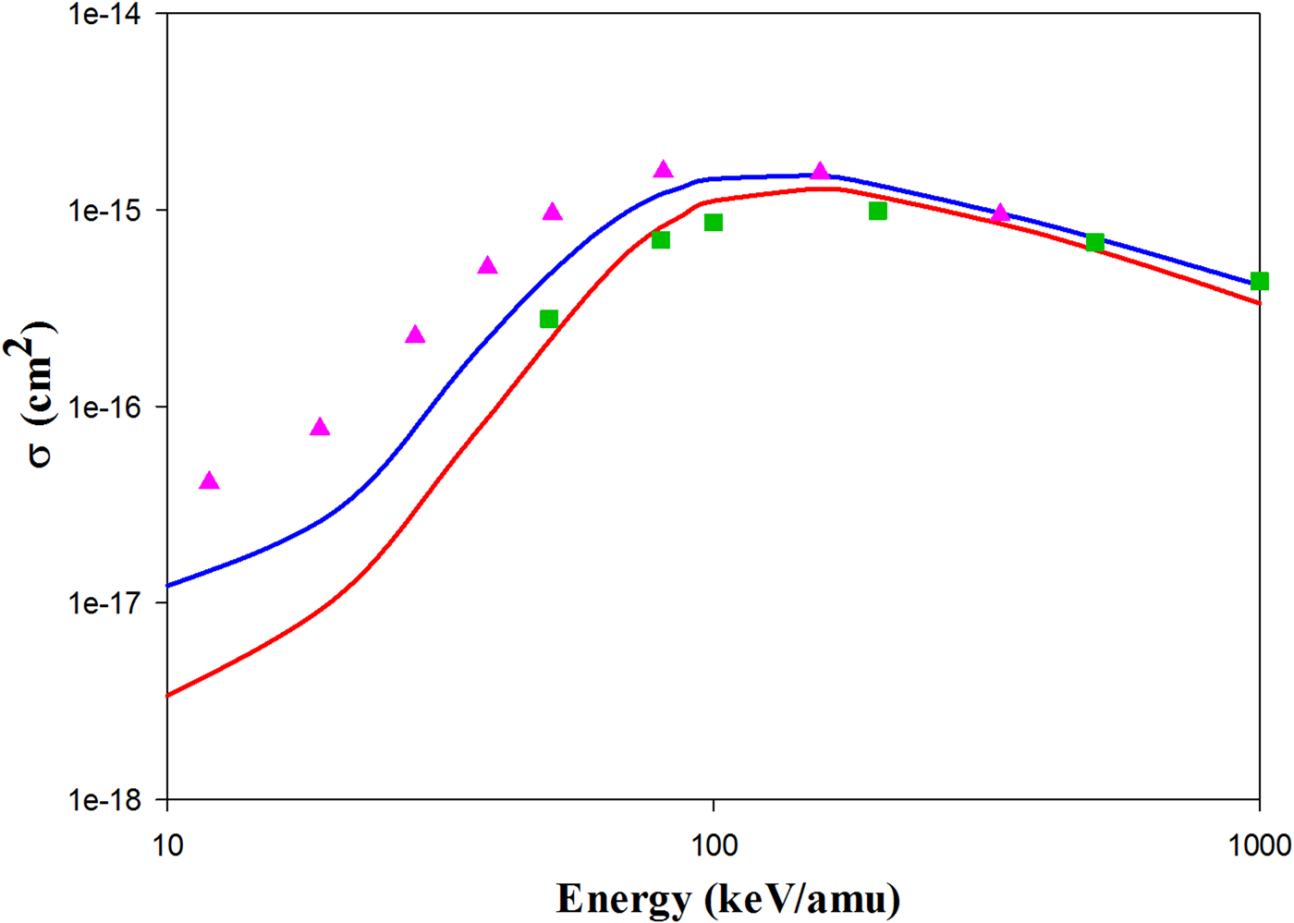
Projectile energy dependent ionization cross sections in B^5+^  + H(1s) collisions. Solid red line: present CTMC results, solid blue line: present QCTMC results, pink triangles: AOCC results of Toshima^5^, green squares: CDW results of Rivarola et al*.*^9^.

**Fig. 6:**
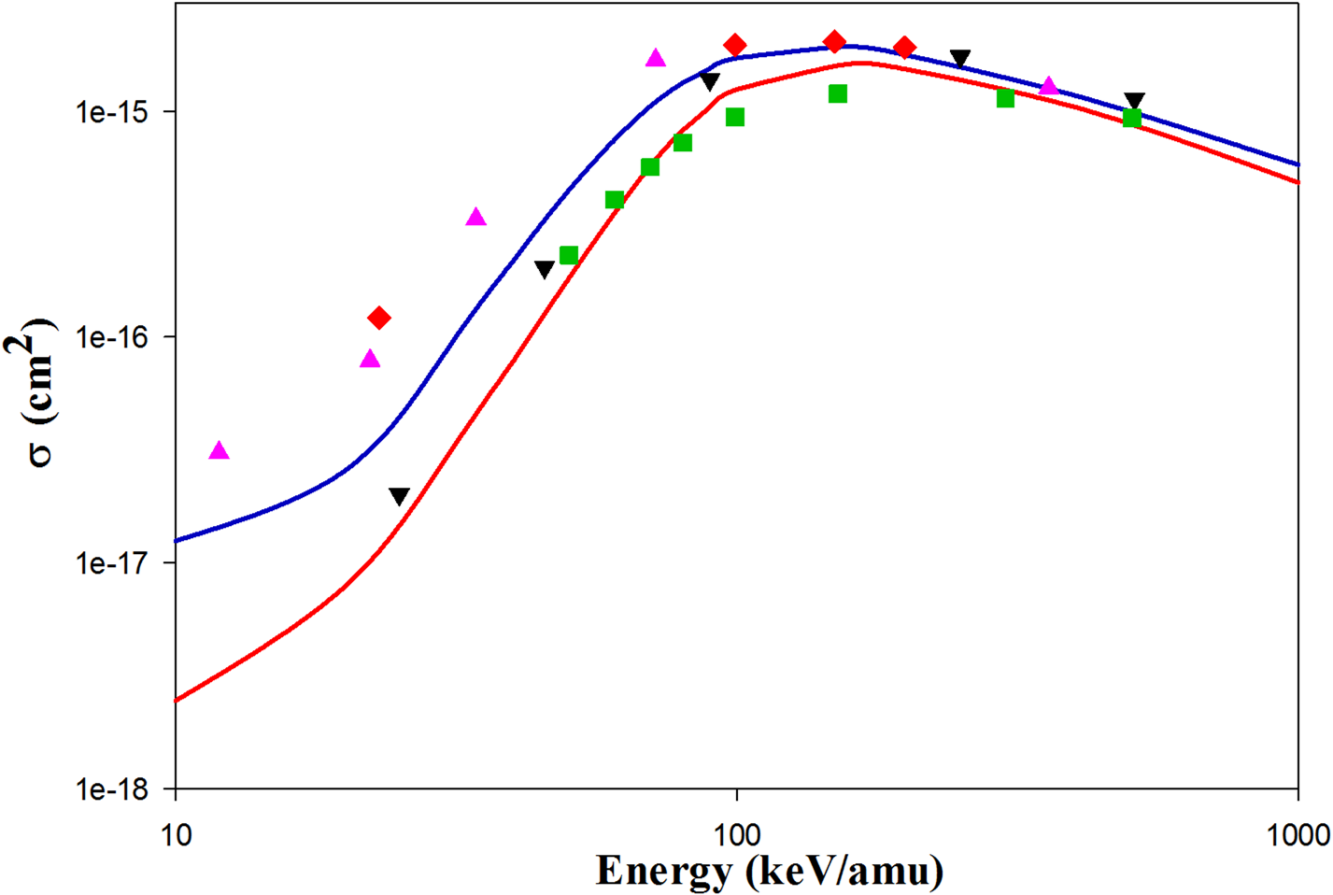
Projectile energy dependent ionization cross sections in C^6+^  + H(1s) collisions. Solid red line: present CTMC results, solid blue line: present QCTMC results, pink triangles: AOCC results of Toshima^5^, green squares: CDW results of Rivarola et al*.*^9^, black inverse triangles: AOCC results of Igenberg et al.^6^, red diamonds: H-CTMC results of Hardie and Olson^23^.

In Figs. 5 and 6, we present the ionization cross sections for B^5+^  + H(1 s) and C^6+^  + H(1 s) collisions, respectively. The higher QCTMC cross sections compared to the CTMC ones result in better agreement between our QCTMC results and the AOCC approaches^5,6^. We also cannot overlook the excellent agreement of our CTMC results with the CDW results of Rivarola^9^. According to Fig. 6, our QCTMC results align well with the hydrogenic-CTMC results of Hardie and Olson^23^.

**Fig. 7:**
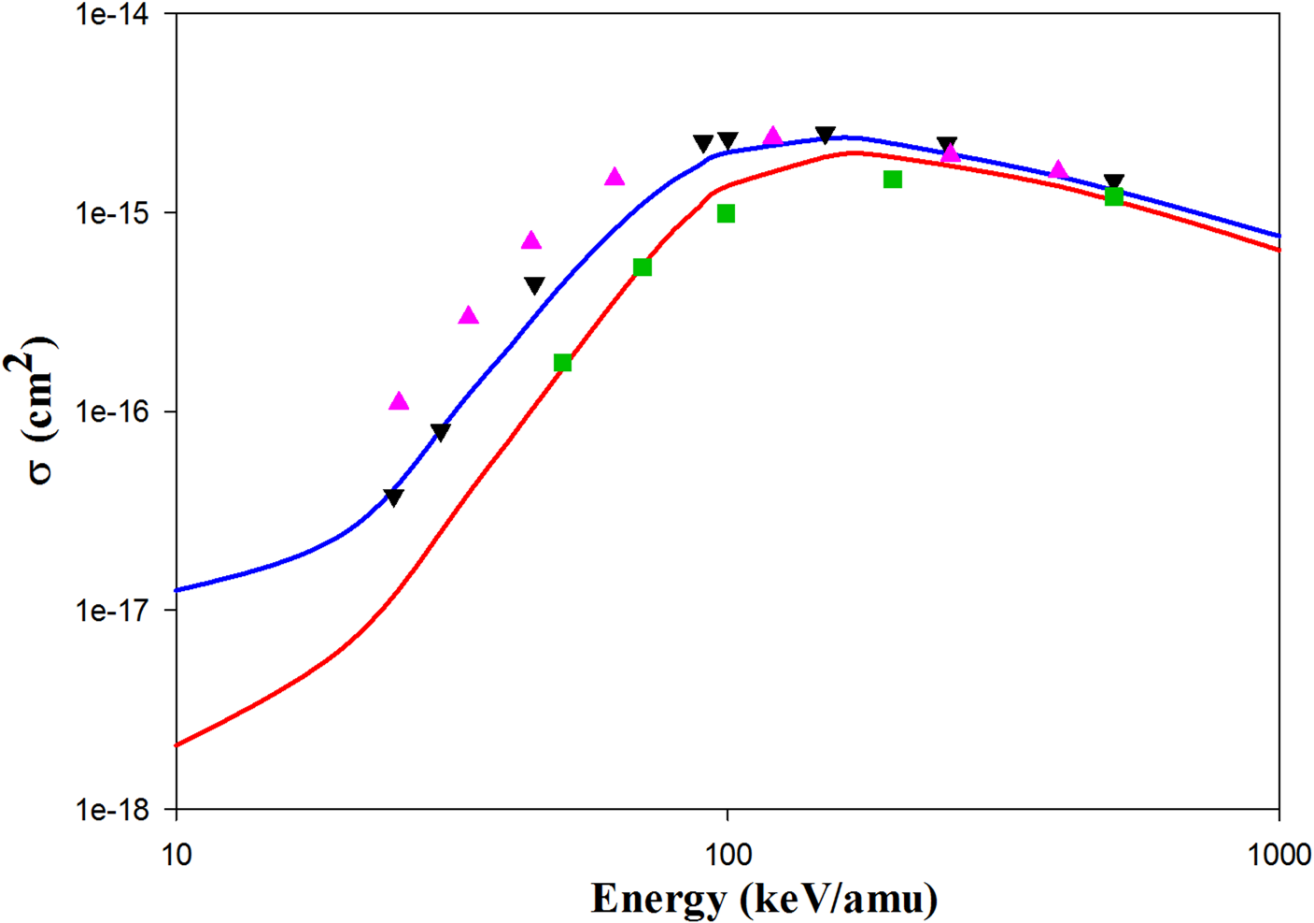
Projectile energy dependent ionization cross sections in N^7+^  + H(1s) collisions. Solid red line: present CTMC results, solid blue line: present QCTMC results, pink triangles: AOCC results of Toshima^5^, green squares: CDW results of Rivarola et al*.*^9^, black inverse triangles: AOCC results of Igenberg et al.^6^.

**Fig. 8:**
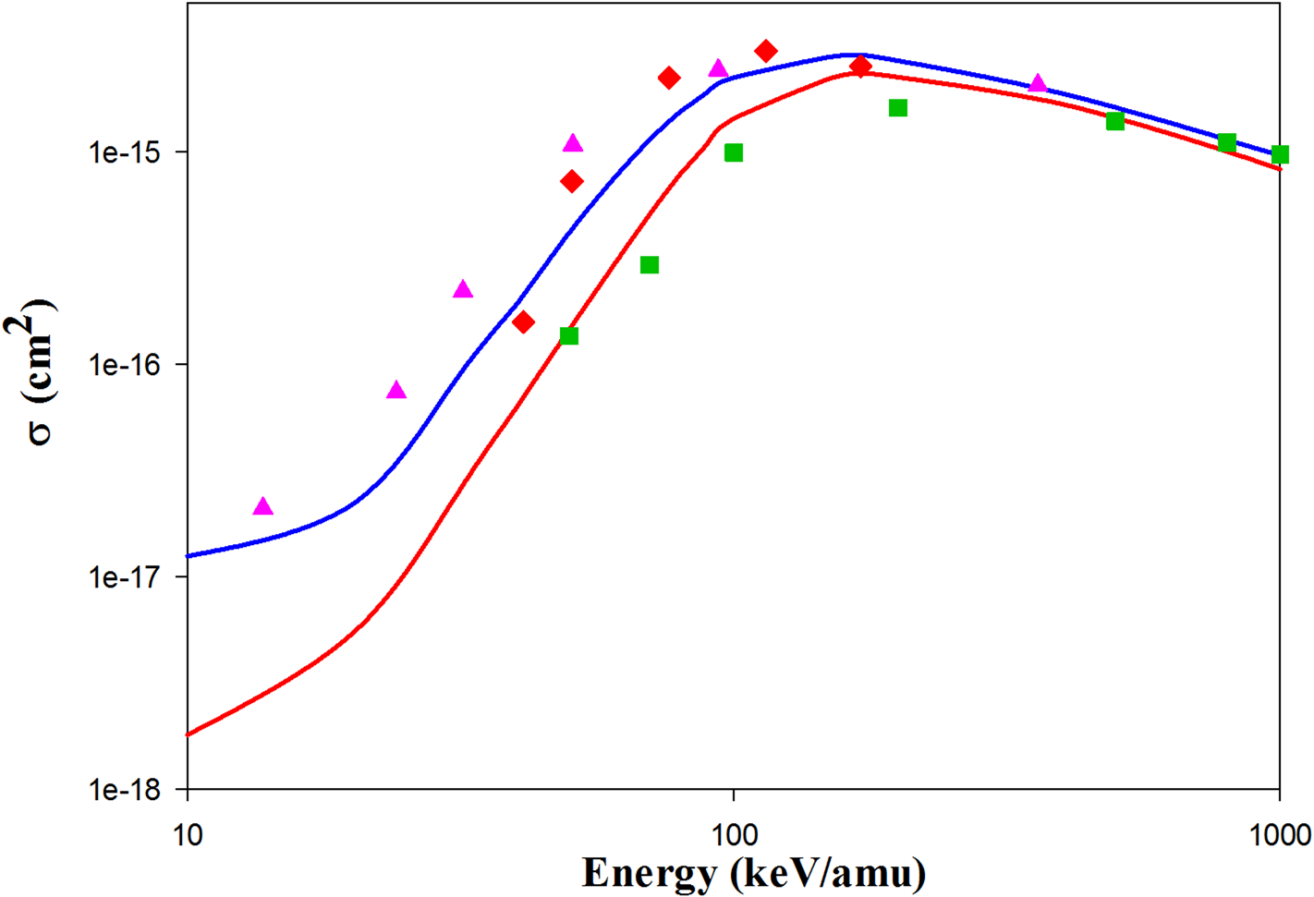
Projectile energy dependent ionization cross sections in O^8+^  + H(1s) collisions. Solid red line: present CTMC results, solid blue line: present QCTMC results, pink triangles: AOCC results of Toshima^5^, green squares: CDW results of Rivarola et al*.*^9^, red diamonds: H-CTMC results of Hardie and Olson^23^.

Figures 7 and 8 present the current CTMC and QCTMC results for ionization cross sections in N^7+^  + H(1s) and O^8+^  + H(1s) as functions of impact energy, respectively. The QCTMC results align closely with the AOCC approaches^5,6^ (see Fig. 5). There is also strong agreement between the present QCTMC results and the AOCC results of Toshima^5^, as well as the hydrogenic-CTMC results of Hardie and Olson^23^ (see Fig. 8).

According to Figs. 1, 2, 3, 4, 5, 6, 7 and 8, the present CTMC and QCTMC results for the ionization cross sections in collisions between A^Z+^ (1 ≤ Z ≤ 8) and ground state hydrogen atoms are presented in Tables 1, 2, 3 and 4. It is evident that the QCTMC ionization cross sections are higher than those of the CTMC. To explain this behavior, we consider the effect of the Heisenberg correction term in the QCTMC model. This term generates a repulsive force that opposes the attractive Coulomb force between the electron and the target nucleus. As a result, the connection between the electron and the nucleus becomes weaker. Therefore, the probability of losing the target electron in the QCTMC model is higher, which leads to increased cross sections in the QCTMC model compared to the CTMC.

**Table 1:** Cross sections for ionization and statistical error values (in parenthesis) from H(1s) by A^Z+^.

A^Z+^	Correction	Energy (keV/amu)			
		10	20	30	40
H^+^	With	1.24E-17 (4.95E-20)	5.46E-17 (8.72E-20)	1.06E-16 (1.18E-19)	1.46E-16 (1.33E-19)
	Without	8.06E-18 (3.23E-20)	3.58E-17 (8.10E-20)	7.67E-17 (1.21E-19)	1.18E-16 (1.50E-19)
He^2+^	With	9.80E-18 (5.70E-20)	3.99E-17 (1.48E-19)	1.17E-16 (2.63E-19)	2.17E-16 (3.30E-19)
	Without	5.72E-18 (3.40E-20)	2.33E-17 (8.14E-20)	6.72E-17 (1.54E-19)	1.46E-16 (2.32E-19)
Li^3+^	With	1.16E-17 (6.97E-20)	3.26E-17 (1.75E-19)	1.14E-16 (3.46E-19)	2.45E-16 (4.87E-19)
	Without	5.32E-18 (3.81E-20)	1.66E-17 (8.46E-20)	5.19E-17 (1.74E-19)	1.32E-16 (2.79E-19)
Be^4+^	With	1.20E-17 (7.95E-20)	2.95E-17 (2.03E-19)	9.92E-17 (4.10E-19)	2.41E-16 (5.99E-19)
	Without	4.20E-18 (3.41E-20)	1.23E-17 (8.22E-20)	3.99E-17 (1.87E-19)	1.08E-16 (3.16E-19)
B^5+^	With	1.22E-17 (8.65E-20)	2.84E-17 (2.17E-19)	9.49E-17 (4.62E-19)	2.46E-16 (7.05E-19)
	Without	3.37E-18 (3.28E-20)	1.02E-17 (8.29E-20)	3.60E-17 (2.06E-19)	9.85E-17 (3.58E-19)
C^6+^	With	1.24E-17 (8.96E-20)	2.55E-17 (2.16E-19)	8.32E-17 (4.74E-19)	2.18E-16 (7.64E-19)
	Without	2.45E-18 (2.87E-20)	7.83E-18 (7.43E-20)	2.79E-17 (1.93E-19)	7.87E-17 (3.55E-19)
N^7+^	With	1.25E-17 (8.97E-20)	2.39E-17 (2.14E-19)	7.89E-17 (4.98E-19)	2.05E-16 (7.95E-19)
	Without	2.08E-18 (2.83E-20)	6.41E-18 (7.10E-20)	2.40E-17 (1.90E-19)	7.03E-17 (3.63E-19)
O^8+^	With	1.24E-17 (7.84E-20)	2.18E-17 (1.72E-19)	7.51E-17 (4.38E-19)	1.91E-16 (8.95E-19)
	Without	1.80E-18 (2.24E-20)	5.38E-18 (5.51E-20)	2.10E-17 (1.58E-19)	6.24E-17 (2.57E-19)

**Table 2:** Cross sections for ionization and statistical error values (in parenthesis) from H(1s) by A^Z+^.

A^Z+^	Correction	Energy (keV/amu)			
		50	60	70	80
H^+^	With	1.70E-16 (1.32E-19)	1.72E-16 (1.36E-19)	1.65E-16 (1.24E-19)	1.62E-16 (1.17E-19)
	Without	1.42E-16 (1.10E-19)	1.51E-16 (1.13E-19)	1.49E-16 (1.11E-19)	1.40E-16 (9.98E-20)
He^2+^	With	3.18E-16 (3.80E-19)	3.84E-16 (3.98E-19)	4.32E-16 (4.01E-19)	4.48E-16 (3.92E-19)
	Without	2.35E-16 (2.76E-19)	3.10E-16 (3.21E-19)	3.59E-16 (3.34E-19)	3.89E-16 (3.19E-19)
Li^3+^	With	4.02E-16 (5.70E-19)	5.56E-16 (6.36E-19)	6.58E-16 (6.36E-19)	7.29E-16 (6.40E-19)
	Without	2.54E-16 (3.73E-19)	3.87E-16 (4.24E-19)	4.99E-16 (4.82E-19)	5.90E-16 (4.83E-19)
Be^4+^	With	4.34E-16 (7.45E-19)	6.47E-16 (8.52E-19)	8.36E-16 (8.97E-19)	9.70E-16 (8.96E-19)
	Without	2.31E-16 (4.38E-19)	3.93E-16 (5.18E-19)	5.65E-16 (6.10E-19)	7.10E-16 (6.55E-19)
B^5+^	With	4.68E-16 (8.91E-19)	7.33E-16 (1.01E-18)	9.91E-16 (1.11E-18)	1.20E-15 (1.16E-18)
	Without	2.15E-16 (4.91E-19)	3.95E-16 (6.32E-19)	6.16E-16 (7.16E-19)	8.16E-16 (8.06E-19)
C^6+^	With	4.42E-16 (1.00E-18)	7.31E-16 (1.18E-18)	1.05E-15 (1.32E-18)	1.33E-15 (1.41E-18)
	Without	1.79E-16 (5.07E-19)	3.43E-16 (7.02E-19)	5.71E-16 (8.09E-19)	8.24E-16 (9.07E-19)
N^7+^	With	4.32E-16 (1.11E-18)	7.40E-16 (1.33E-18)	1.10E-15 (1.51E-18)	1.46E-15 (1.61E-18)
	Without	1.61E-16 (5.39E-19)	3.14E-16 (7.20E-19)	5.37E-16 (8.84E-19)	8.21E-16 (1.00E-18)
O^8+^	With	4.16E-16 (1.25E-18)	7.36E-16 (1.51E-18)	1.13E-15 (1.66E-18)	1.55E-15 (1.83E-17)
	Without	1.45E-16 (4.01E-19)	2.87E-16 (5.49E-19)	5.04E-16 (6.81E-19)	7.92E-16 (1.12E-18)

**Table 3:** Cross sections for ionization and statistical error values (in parenthesis) from H(1s) by A^Z+^.

A^Z+^	Correction	Energy (keV/amu)			
		90	100	150	200
H^+^	With	1.58E-16 (1.08E-19)	1.47E-16 (1.03E-19)	1.10E-16 (8.19E-20)	8.93E-17 (7.03E-20)
	Without	1.31E-16 (9.57E-20)	1.23E-16 (9.22E-20)	8.98E-17 (7.16E-20)	6.90E-17 (6.17E-20)
He^2+^	With	4.46E-16 (3.71E-19)	4.32E-16 (3.45E-19)	3.72E-16 (2.95E-19)	3.01E-16 (2.43E-19)
	Without	3.91E-16 (3.10E-19)	3.92E-16 (3.03E-19)	3.21E-16 (2.42E-19)	2.58E-16 (2.13E-19)
Li^3+^	With	7.61E-16 (6.04E-19)	7.68E-16 (6.10E-19)	7.04E-16 (5.25E-19)	5.97E-16 (4.24E-19)
	Without	6.30E-16 (4.82E-19)	6.66E-16 (4.81E-19)	6.18E-16 (4.12E-19)	5.25E-16 (3.65E-19)
Be^4+^	With	1.05E-15 (8.98E-20)	1.11E-15 (8.86E-19)	1.09E-15 (7.23E-19)	9.55E-16 (6.40E-19)
	Without	8.19E-16 (6.51E-19)	9.02E-16 (6.60E-19)	9.48E-16 (6.07E-19)	8.40E-16 (5.17E-19)
B^5+^	With	1.34E-15 (1.14E-18)	1.43E-15 (1.10E-18)	1.49E-15 (9.84E-19)	1.33E-15 (8.77E-19)
	Without	9.83E-16 (8.20E-19)	1.11E-15 (8.39E-19)	1.27E-15 (7.97E-19)	1.17E-15 (6.93E-19)
C^6+^	With	1.56E-15 (1.42E-18)	1.71E-15 (1.43E-18)	1.92E-15 (1.24E-18)	1.77E-15 (1.08E-18)
	Without	1.06E-15 (9.77E-19)	1.24E-15 (1.01E-18)	1.58E-15 (9.22E-19)	1.53E-15 (8.51E-19)
N^7+^	With	1.76E-15 (1.68E-18)	2.00E-15 (1.71E-18)	2.34E-15 (1.59E-18)	2.21E-15 (1.36E-18)
	Without	1.11E-15 (1.11E-18)	1.35E-15 (1.17E-18)	1.89E-15 (1.16E-18)	1.89E-15 (1.04E-18)
O^8+^	With	1.93E-15 (1.93E-18)	2.23E-15 (1.98E-18)	2.78E-15 (1.79E-18)	2.67E-15 (1.62E-18)
	Without	1.11E-15 (1.23E-18)	1.42E-15 (1.28E-18)	2.20E-15 (1.34E-18)	2.23E-15 (1.25E-18)

**Table 4:** Cross sections for ionization and statistical error values (in parenthesis) from H(1s) by A^Z+^.

A^Z+^	Correction	Energy (keV/amu)			
		300	400	800	1000
H^+^	With	6.24E-17 (6.88E-20)	4.80E-17 (5.46E-20)	2.70E-17 (4.09E-20)	2.26E-17 (3.74E-20)
	Without	4.74E-17 (6.04E-20)	3.56E-17 (5.16E-20)	1.80E-17 (3.22E-20)	1.45E-17 (2.85E-20)
He^2+^	With	2.21E-16 (2.09E-19)	1.75E-16 (1.42E-19)	9.56E-17 (9.36E-20)	8.00E-17 (8.50E-20)
	Without	1.81E-16 (1.84E-19)	1.39E-16 (1.34E-19)	7.12E-17 (8.76E-20)	5.72E-17 (7.21E-20)
Li^3+^	With	4.46E-16 (3.17E-19)	3.53E-16 (2.75E-19)	1.97E-16 (1.72E-19)	1.67E-16 (1.47E-19)
	Without	3.83E-16 (2.77E-19)	2.99E-16 (2.29E-19)	1.56E-16 (1.43E-19)	1.25E-16 (1.27E-19)
Be^4+^	With	7.36E-16 (4.93E-19)	5.88E-16 (4.12E-19)	3.36E-16 (2.65e-19)	2.83E-16 (2.13E-19)
	Without	6.47E-16 (4.14E-19)	5.12E-16 (3.62E-19)	2.73E-16 (2.19E-19)	2.22E-16 (1.85E-19)
B^5+^	With	1.05E-15 (6.91E-19)	8.52E-16 (5.60E-19)	4.95E-16 (3.68E-19)	4.13E-16 (2.99E-19)
	Without	9.29E-16 (5.62E-19)	7.51E-16 (4.72E-19)	4.13E-16 (3.06E-19)	3.35E-16 (2.73E-19)
C^6+^	With	1.41E-15 (9.19E-19)	1.17E-15 (7.63E-19)	6.91E-16 (4.50E-19)	5.79E-16 (4.04E-19)
	Without	1.25E-15 (7.35E-19)	1.04E-15 (6.26E-19)	5.91E-16 (4.12E-19)	4.83E-16 (3.52E-19)
N^7+^	With	1.82E-15 (1.17E-18)	1.51E-15 (9.19E-19)	9.04E-16 (5.77E-19)	7.59E-16 (4.97E-19)
	Without	1.60E-15 (8.82E-19)	1.34E-15 (7.22E-19)	7.84E-16 (5.31E-19)	6.45E-16 (4.55E-19)
O^8+^	With	2.24E-15 (1.41E-18)	1.87E-15 (1.15E-18)	1.13E-15 (7.47E-19)	9.59E-16 (6.73E-19)
	Without	1.94E-15 (1.06E-18)	1.67E-15 (9.31E-19)	1.00E-15 (6.64E-19)	8.26E-16 (5.48E-19)

On the other hand, due to the extended interaction time, quantum mechanical effects cannot be ignored at low impact energies. Consequently, the QCTMC results align more closely with quantum mechanical approaches than the CTMC results at low impact energies (see Figs. 1, 2, 3, 4, 5, 6, 7 and 8). This difference gradually decreases with increasing projectile energy.

## Conclusions

The ionization cross sections in A^Z+^ for 1 ≤ Z ≤ 8 and the ground state hydrogen atom have been presented within the framework of the CTMC and QCTMC models. To enhance the accuracy of the cross sections, we calculated 5 × 10^6 individual trajectories in both the CTMC and QCTMC models for each impact energy. Our classical results were compared with previous quantum mechanical approaches such as AOCC, CDW, and available experimental data. We found that the QCTMC cross sections are consistently higher than the CTMC cross sections across the entire range of impact energies, with a significant difference observed at low projectile energies. By adding a correction term to account for the Heisenberg uncertainty principle in the classical Hamiltonian, we demonstrated that the QCTMC ionization cross sections align well with both quantum mechanical results and experimental data, particularly at low impact energies. We believe that the quasi-classical trajectory Monte Carlo model significantly improves the computed ionization cross sections, allowing for accuracy comparable to quantum–mechanical results through simpler calculations.

## Data Availability

The datasets used and/or analysed during the current study available from the corresponding author on reasonable request.
